# Electron energization dynamics in interaction of self-generated magnetic vortices in upstream of collisionless electron/ion shocks

**DOI:** 10.1038/s41598-022-11163-2

**Published:** 2022-05-05

**Authors:** N. Naseri, S. G. Bochkarev, V. Y. Bychenkov, V. Khudik, G. Shvets

**Affiliations:** 1 Institute for Fusion Studies and Department of Physics, The University of Texas, 1 University Station C1500, Austin, 78712 TX USA; 2 Department of Physics and Astronomy, Middle Tennessee State University, Wiser-Patten Science Hall, 422 Old Main Cir, Murfreesboro, 37132 TN USA; 3 P.N. Lebedev Physics Institute of the Russian Academy of Sciences, Moscow, 119991 Russia; 4 School of Applied and Engineering Physics, Cornell University, Ithaca, NY 14850 USA

**Keywords:** Astronomy and astrophysics, Plasma physics

## Abstract

Relativistic collisionless shocks are considered responsible for particle energization mechanisms leading to particle acceleration. While electron energization in shock front region of electron/ion collisionless shocks are the most studied, the mechanism of electron energization in interaction with self-generated magnetic vortices (MVs) in the upstream region is still unclear. We investigate electron energization mechanism in the upstream region of electron/ion relativistic collisionless shocks, using two dimensional particle-in-cell (PIC) simulations. We discuss mechanism of electron energization which takes place in the upstream region of the shock, where the counter stream particles interact with incoming flow. The energy gain of electrons happens during their interaction with evolving fields of self-generated magnetic vortices in this region. Three Fermi-like electron energization scenarios are discussed. Stochastic acceleration of electrons in interaction with fields of MV leads to anisotropic heating of fast electrons due to diffusion in the momentum space of electrons and, finally, synergetic effect of evolving fields of MVs leads to the formation of a power-law tail of supra-thermal particles.

## Introduction

Collisionless shocks are unique phenomena in space and astrophysical plasma environment such as supernova remnants, gamma-ray bursts, active galactic nuclei, and binary systems. One of the key features in astrophysical collisionless shocks is particle acceleration.The energized particles either escape the acceleration region and become Cosmic Rays (CR), and/or they interact with ambient backgrounds to produce high energy photons. The spectrum of the radiation emitted by high energy particles from indirect observations and by measurements of CR and gamma-ray bursts (GRBs) spectra show evidences of non-thermal particle acceleration generated by collisionless shocks^1–3^. These observations as well as numerical simulations of unmagnetized relativistic collisionless shocks have shown the evidence of electron energization in shock front along with electron energization in the upstream region of the electron/ion shock^1,2,4–7^. While electron energization in shock front region of collisionless shocks are the most commonly studied, electron energization in other large-scale regions of the shocks (e.g. in the upstream region) is of great interest. The focus of this study is on electron energization and acceleration mechanism during interaction with self-generated magnetic vortices in the upstream of relativistic collisionless electron/ion shocks with no external magnetic field.

Collisionless shocks are believed to be mediated by Weibel instability which leads to fast growth of magnetic field at small scales, plasma isotropization and particle energization at later times^6,8–13^. A first phase of magnetic field amplification due to Weibel instability happens in the shock front region^6,14,15^. A simultaneous stage of magnetic field growth happens due to development of Weibel instability driven by particles moving ahead of shock front, i.e counter streams, and the incoming cold plasma streams. Counter stream electron flow are electrons that are either reflected off shock transition region or escaped interaction with shock transition region. In addition to Weibel instability, Biermann battery^16^ induced by nonparallel temperature and plasma density gradients, leads to generation of spontaneous nonlinear magnetic modes such as monopole and dipole vortices (see e.g.^17,18^).

Recently we demonstrated that magnetic vortices (MVs) can self-consistently emerge in the upstream of electron/ion collisionless shocks with no external magnetic field^19^. The early stage of the interaction involves the generation of quasi-linear elongated MVs, which subsequently merge to form circularly shaped MVs (bubble). Localized regions of the strong magnetic field in the form of magnetic dipole vortices upstream of the shock were observed in the simulations developed during the nonlinear evolution of the electron and ion filaments.

The magnetic vortex generation occurs at the stage where flow energy transformation into thermal energy takes place^19^. The MV’s magnetic (and electric) fields grow, and their longitudinal size shrink as they move towards shock front after formation. Considerable part of ion kinetic energy is finally converted into the thermal energy ($13\%$). At the same time, a small group of particles (both electrons and ions) are accelerated to high energies more than the initial kinetic energy of flow particles. The electron and ion energy distributions in the vortex domain have considerable nonthermal parts, which confirms the energy dissipation of the bulk ion beam.

Most previous works investigated electron energization in interaction with magnetic structure in shock front region^6,7^. In shock front region, electrons gain energy through Fermi acceleration mechanism^20–25^ by crossing the shock front back and forth. The energy of the electrons increases with time as the number of crossings increases. Other mechanisms considered in the literature (see e.g.,^26–28^) are stochastic particle acceleration and acceleration by direct electric fields. These acceleration processes develop not only at shocks, but in reconnection events^29,30^. In this paper, we focus on electron energization processes, which takes place not in the shock front, but in the upstream region of the shock, where the counter stream particles interact with incoming flow. The energy gain of electrons happens only during interaction of electrons with evolving fields of MVs. The MVs annihilate before reaching the shock front^19^. We identified three scenarios of Fermi-like electron energization and correspondingly three group of energized electrons in interaction with evolving fields of self-generated magnetic vortices in the upstream region of the shock. First and second scenarios correspond to energization of counter stream electrons. These electrons move towards overlapping region, where the cold fresh incoming flows meet the counterstream flows. These electrons can gain significant amount of energy through kick-like stochastic energization process^28,31^, where particles receive kicks due to interaction with evolving MV fields with steep gradients. The third scenario identified in our simulations is energization of incoming electron flow. The incoming electrons can trap in the fields of MVs in the very early stage of their formation. They move with MV towards shock front region and leave MVs in their final stage of MV, while gaining energy in the process of stochastic acceleration in evolving fields of MV.

Tracking a large number of energized electrons from the non-thermal tail of the energy spectrum shows that particles in this region are directly accelerated by the large inductive transverse and longitudinal electric fields of the evolving MVs in this region. The evolution of energy distribution for the upstream region shows a power-law tail of supra-thermal particles, which saturates rather quickly after the shock forms and stay stable with power law index of $p \approx 2.1$.

The paper is organized as follows: simulation set-up is the first section, followed by self-generated MVs formation mechanism in the upstream, MV structure and non-thermal electron tail formation in the upstream. Then we present scenarios of electron energization in interaction with self-generated MVs. The last section includes discussion and conclusion.

## Results

In this section, we present the results of a 2D PIC simulation of the formation and evolution of MWs, as well as the electron energization in a collisionless electron-ion shock.

### Simulation set-up

The two-dimensional (2D) version of the relativistic parallel particle-in-cell (PIC) code VLPL is used^32^. The code was modified to minimize noise properties of numerical instabilities, by using third-order shaped particles and current smoothing. A rectangular simulation box in the $x-z$ plane with the dimensions $L_x = 1300~l_{pe}$ and $L_z = 130~l_{pe}$ and the grid sizes $\Delta z = l_{pe}/10$ and $\Delta x = l_{pe}/10$ is used. Here $l_{pe}=c/\omega _{pe}$ is the electron inertial length, that is the typical transverse spacial scale of the filaments, $\omega _{pe}=\sqrt{\frac{4\pi n_0e^2}{\gamma _0 m_e}}$ is the electron plasma frequency, $\gamma _0$ is the relativistic gamma factor of incoming plasma flow, *e* and *m* denote the charge and mass of electron, and $n_0$ is the unperturbed density of electrons. Periodic boundary conditions are applied for particles and fields in the transverse (*z*) direction. The left boundary is reflecting for particles and fields. Simulation stops when counterstream particles reach the right boundary of the simulation domain. Therefore, no particle leaves the domain during simulation. Fresh flow of electrons and ions enter the domain continuously from the right boundary. Each computational cell is initialized with 16 macro-particles: 8 electrons and 8 ions. We assume that initially the electron-ion (e,i) plasma beam with the mass ratio $m_i/m_e=32$ and equal charges $q_i=q_e$, equal densities $n_0$, and relativistic velocities $v_x$ (corresponding to $\gamma _0 \equiv 1/\sqrt{1-v_x^2/c^2} = 15$) moves to the left (in the direction opposite to *x*-axis direction). To reduce the computational effort, the initial contact point of the two counter-propagating streams is modeled as a reflecting wall at $x=0$ ^6^. The simulation is performed in the reflecting wall frame, where the downstream (thermalized) plasma behind the shock has a vanishing average flow velocity. All densities (electron and ion) and fields (electric and magnetic) are expressed in dimensionless units as $\tilde{N}_{i,e} = n_{i,e}/n_0$, $\tilde{B}_y = eB_y/m_e\omega _{pe}c\sqrt{\gamma _0}$, and $\tilde{E}_{x,z} = eE_{x,z}/m_e\omega _{pe}c\sqrt{\gamma _0}$.

**Figure 1:**
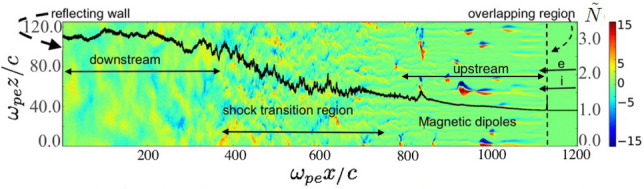
Schematic of the interaction of two interpenetrating electron/ion beams: Color plot of normalized out of plane magnetic field ($B_y$) in $x-z$ plane at $\omega _{pe}t=1140$. Solid black line (axis on right): transversely averaged density $\tilde{N}$ normalized to upstream unperturbed density $n_0$. The dashed line is where the incoming e/i beams meet the counter streams.

### MV formation and structure

The structure of the fully formed shock at $\omega _{pe}t=1140$ is shown in Fig. 1. The transversely averaged density $\tilde{N}(x)=\langle n(x,z)/n_0 \rangle$ (black line; $\langle \rangle$ denotes transverse averaging over the *z*-dimension) and the color plot of the normalized transverse magnetic field ($B_y$) are plotted in Fig. 1 that was chosen to represent a fully developed shock.

**Figure 2:**
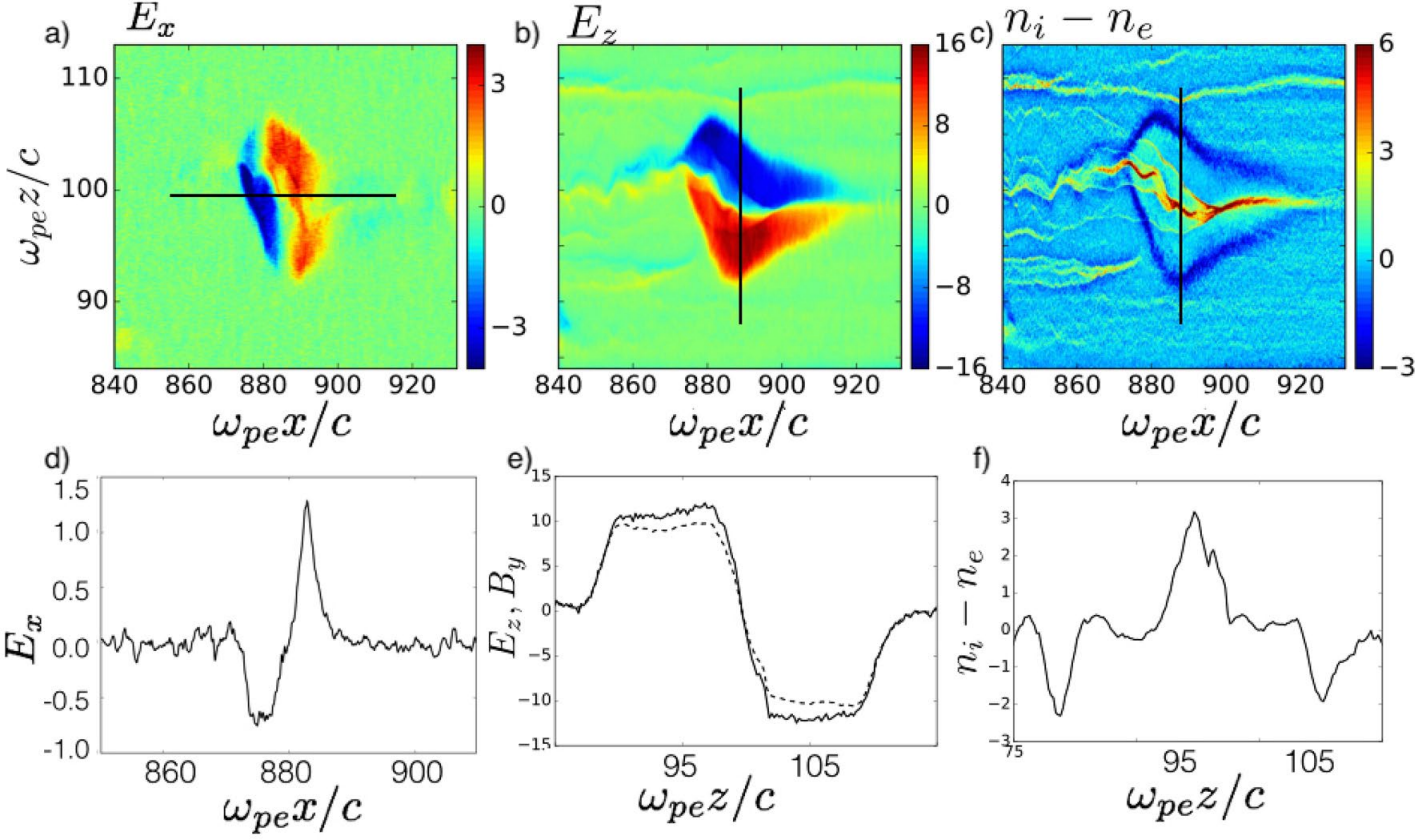
(**a**) Distribution of longitudinal electric field ($E_x$) of MV, (**b**) transverse electric field ($E_z$) (**c**) charge density at $\omega _{pe}t=1057$. (**d**) lineout of the longitudinal electric field along line shown in (**a**). (**e**) lineouts of the transverse electric field (dashed line) and out of plane magnetic field (solid line) corresponding to the line shown in (**b**). (**f**) lineout of charge density along the line shown in (**c**).

**Figure 3:**
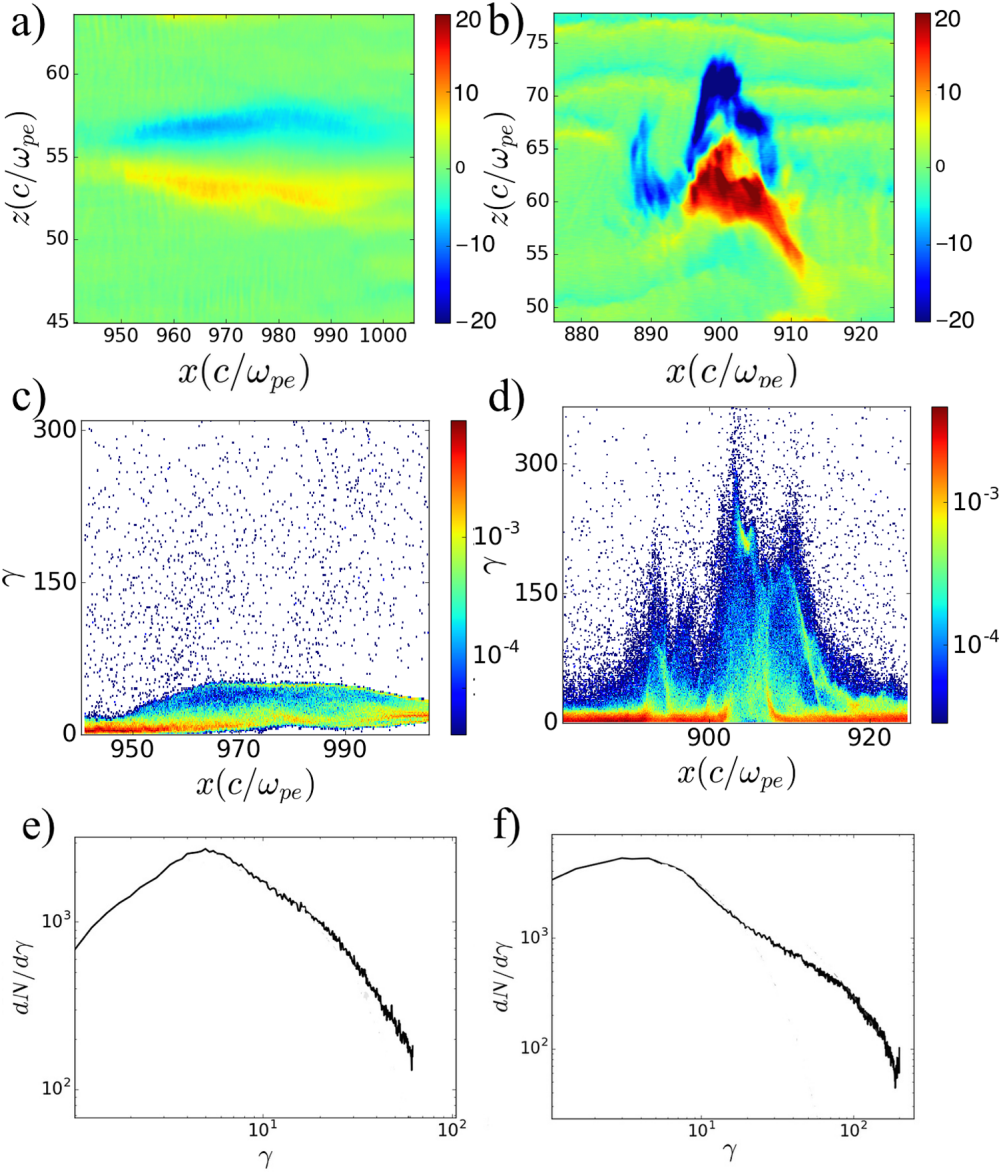
(**a**,**b**) Magnetic field ($B_y$) distribution evolution of a typical MV at $\omega _{pe}t=1038$ and 1178. (**c**,**d**) electron energy distributions averaged over transverse length of MV. (**e**,**f**) electron energy spectrum evolution corresponding to $\omega _{pe}t=1038$ and 1178 averaged over longitudinal and transverse size of MV.

Our focus is on the upstream region of the shock, where the incoming cold streams interact with isotropized counter stream flow. The upstream region of the relativistic collisionless shocks is dominated by the so-called counter stream particles, incoming flow of particles interacting with lower density stream of hot isotropized counter stream electrons and relatively cold counter stream ions, that already escaped the shock region or were never trapped. Here we summarize MV generation and evolution in the upstream. In overlapping region, the incoming cold streams of electrons and ions meet with counter stream isotropized electrons and ions with considerable longitudinal and perpendicular velocity spreads (thermal spread). The interaction of counter stream flow with cold incoming flow leads to electron Weibel instability. However, due to thermal spread, the growth rate of electron Weibel instability is low as compared to that for cold beam plasma ($\delta _e\simeq \sqrt{2} \omega _{pe} v_x/c\sim \omega _{pe}$, where $\delta _e$ is the growth rate of electron Weibel instability)^33^. Electron Weibel instability initiates quickly as the incoming and counter streams meet in the overlapping region. The growth rate of electron Weibel instability is found to be $0.13~\omega _{pe}$ that is significantly less than for the cold beam plasma case $\delta _e=\sqrt{2}~\omega _{pe}$, and in agreement with the estimate for a hot electron beam $\simeq \delta _e (1-\Delta \gamma _{\perp }/\gamma _0)$^9^, where $\Delta \gamma _{\perp }$ is the transverse energy spread and $\Delta \gamma _{\perp } \backsimeq \gamma _0$. Initially, small-scale filaments are formed, magnetic field grows and then instability saturates. The maximum value of generated magnetic field is in agreement with estimate for saturation level $B_{y,s}\simeq \sqrt{\gamma _0}\,$^34^. There is no considerable charge imbalance in filaments as electron Weibel instability saturates in overlapping region, where the counter stream flow meets cold flow^19^. At the final stage of electron Weibel instability, the electrons in incoming beam are considerably isotropic. The ion Weibel instability is later developed in the background of well-thermalized electrons. The growth rate of ion Weibel instability is found to be close to $0.34~\omega _{pi}$, (here, $\omega _{pi}= \sqrt{4\pi e^2 n_0 /\gamma _0 m_i}$ is the ion plasma frequency) that is less than the estimate for cold approximation ($\delta _i=\sqrt{2}~\omega _{pi}$)^15^, since there is considerable background magnetic field that makes a standard linear analysis not applicable. Current pinching and filament neck formation (see Fig. 2c below) happens as linear stage of ion Weibel instability terminates, which as mentioned results in magnetic field growth and more pinching which leads to formation of elongated localized magnetic vortices, and their transformation into large-scale magnetic bubbles. Figure 3a,b show the distributions of the out of plane plane magnetic fields ($B_y$) at the initial (linear) stage of the MV generation at $\omega _{pe}t=1038$ and the saturated nonlinear stage of MV at $\omega _{pe}t=1178$. We typically observed magnetic field enhancement of the MVs by a factor of $\approx 5 \sim \sqrt{m_i/m_e}$ with respect to the background magnetic field while they propagate towards the shock front^19^. The size of MVs grow to $c/\omega _{pi}$. Reduced ion to electron mass ratio affects the structure dynamics. An evident example is electron-positron plasma. In our high-resolution simulations we used $m_i/m_e=32$ due to computational constraints which made it possible to shed light on the physics of the process. However without using realistic ion to electron mass ratio, we cannot fully predict plasma evolution at a long stage.

Figure 2a,b shows the structure of longitudinal and transverse electric fields ($E_x$,$E_z$) of nonlinear stage of the MV $\omega _{pe}t=1057$, where the MV is developed but not annihilated yet. Figure 2d–f show the lineouts of the fields and charge density along black overlaid lines shown on top panel. Strong electrostatic electric field is induced around the cavity because of electron evacuation. This field tends to drag the counter stream electrons into the MV. The transverse electric and magnetic fields ($E_z$ and $B_y$) are larger than the longitudinal electrostatic electric field ($E_x$) as can be seen from Fig. 2d,e, however, a combination of both electrostatic and transverse fields determines the entire process of electron energization. The strong Lorentz force $q\mathbf{v} \times \mathbf{B}$, focuses the incoming ion beam, while expelling the incoming electron beam form the center of MV as shown in Fig. 2c. The ion currents are pinched in the self-generated magnetic field. The counter-streaming electron flow follows the ion flow to partly neutralize the beam plasma. However, at the strongly nonlinear stage, significant charge separation appears (see charge density in Fig. 2c). The transverse electric field balances the Lorentz force, $E_z\approx v_xB_y/c$. The ion filament pinching results in an increase in the magnetic field and a consequent increase in the Lorentz force, which can be seen in Fig. 2b,c and corresponding lineouts. The magnetic field of the MV grows during propagation towards downstream. The magnetic field of the MV increases in a non-monotonous manner by a factor of $\approx 5 \sim \sqrt{m_i/m_e}$ with respect to the background magnetic field^19^.

Self-generated MVs move in the plasma density gradient direction toward the shock front^35^. Our simulations show that the axial velocity of the magnetic vortices increases as they move along the density gradient. The drift velocity of magnetic vortices, propagating in a plasma with a density gradient, is estimated to be $v_A/c\approx \sqrt{\frac{\alpha B_y^2}{1+\alpha B_y^2}}$^36^, where $\alpha =\frac{m_en_0}{m_i\gamma _0 n}$; here, *n* is the ion density. The longitudinal drift velocity of the magnetic vortices during evolution observed in simulations increases from $|v_x/c|\approx 0.2$ to 0.5, when the ion density increases from $n_0$ to $\approx 1.5n_0$, and $B_y$ increases from 3.1 to 15. Alfvén velocity corresponding to the ion densities and magnetic field amplitudes taken from simulation leads to a value of $v_A/c\approx 0.14$ to 0.51, in agreement with the observed vortex velocities in our simulations. Eventually, when the counter stream flows contribute to the total plasma density in the region, the drift of MVs slows down as dissipation begins and MVs fields decay. This process is periodic, i.e., vortices appear and disappear after time interval of the order of $50\omega _{pi}^{-1}$.

Figure 3c,d illustrate the electron energy distributions (corresponding to Fig. 3a,b, averaged over transverse size of MV along $z-$ direction). We can see that the electrons gain a large amount of energy in nonlinear stage of MV. At this time, the transverse electric (and magnetic) field of the MV reaches its maximum. The electron energy has its maximum around the center of the MV and decreases with distance from the center.

The low energy part of the electron energy spectrum is fitted by Maxwell-Jüttner distribution $dN/d\gamma =A \gamma \exp (-m_e c^2\gamma /T_e)$, where *A* is the constant, with $T_e \approx 2 m_ec^2$ at $\omega _{pe} t = 1038$ and $T_e \approx 3 m_ec^2$ at $\omega _{pe}t = 1178$. A non-thermal component with energies up to 200 appears at $\omega _{pe}t = 1178$. Figure 3f shows formation of a broad energy distribution that is a superposition of the Maxwell-Jüttner distribution and a plateau, slowly varying energy before fast drop at cutoff.

The upstream region of the shock is characterized by nonparallel temperature and density gradients, driving the Biermann battery mechanism. Since the electron temperature gradient (along *x*-direction) is not steep ($0.01 \omega _{pe}/c$), and the transverse density gradient (along *z*) is of the order of inverse dipole size $0.1\omega _{pe}/c$), the upper limit estimate of the magnetic field growth rate due to the Biermann mechanism ($\frac{\partial \mathbf{B}}{\partial t}$= $-c\nabla T_e \times \nabla n/en$^16^) is an order of magnitude less than magnetic field growth rate of Weibel instability. Note 2D kinetic simulation describes all the causes of MV generation, including the Biermann battery mechanism.

**Figure 4:**
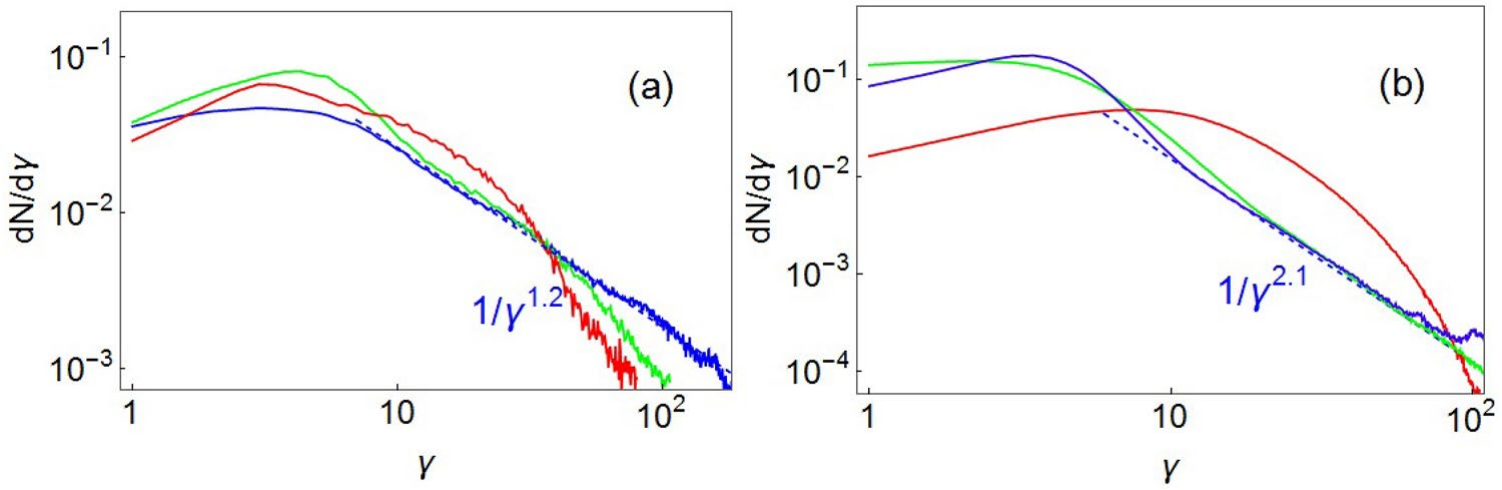
(**a**) Evolution of energy spectrum averaged over longitudinal and transverse size of MV for $\omega _{pe}t$ = 1038 (red), 1178 (green) and 1204 (blue) and corresponding power-law fits (dashed line), ($dN/d\gamma \sim \gamma ^{-p}$), $p\approx 1.2$. (**b**) Evolution of energy spectrum for the entire upstream region for $\omega _{pe} t=162$ (red), 584 (green), 1233 (blue) and corresponding fit (with power index $p\approx 2.1$) for $\omega _{pe}t=1233$

The energy evolution of non-thermal electrons from the tail of spectra averaged over a box enclosing MV, changes with time due to stochastic character of kick-like electron acceleration in electric and magnetic fields of evolving MV (see next section). The energy spectra, averaged over a box enclosing MV, is shown in Fig. 4a. The power-law indices of the non-thermal electron tail distribution varies with time and found to be $p = 1.2$ ($dN/d\gamma \propto \gamma ^{-p}$) corresponding to $\omega _{pe} t = 1204$. No further acceleration happens as the sample MV is dissipated after this time. The evolution of energy distribution for the entire upstream region is shown in Fig. 4b. MVs begin to appear while the shock is forming ($\omega _{pe}t\approx 162$), the longitudinal region where dipoles are located at this time is $\Delta x \approx 36.5 c/\omega _{pe}$. At this time the electrons in this region are thermalized, and the distribution function is close to the Maxwell-Jüttner distribution with $T_e \approx 7 m_e c^2$, no tail formation has yet been observed (Fig. 4-$\omega _{pe}t=162$). After the shock is formed ($\omega _{pe}t=420$), the upstream forms and expands, a power-law of supra-thermal particles is formed, which saturates rather quickly by $\omega _{pe}t \approx 584$ and stay stable with power law index of $p\approx 2.1$. Note that shock front is not a part of the upstream.

**Figure 5:**
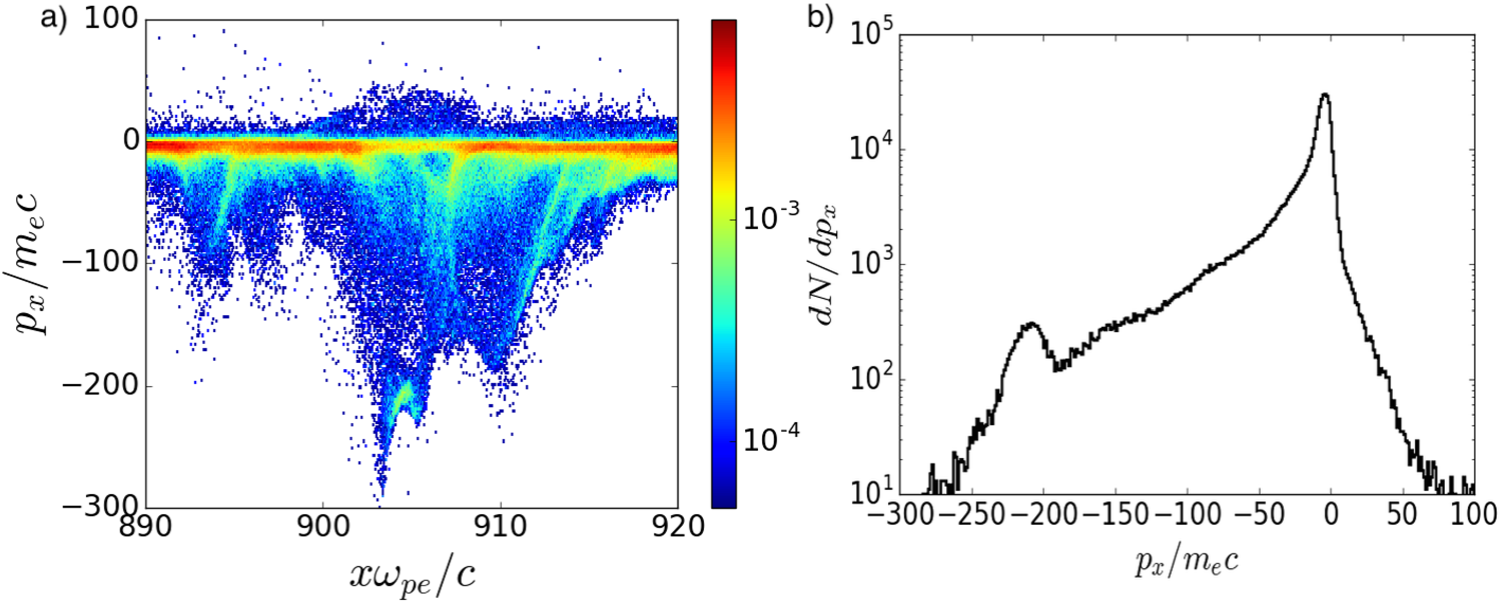
(**a**) electron momentum distribution $p_x/m_ec$ along *x*-direction averaged over transverse size of the MV. (**b**) electron phase spectrum corresponding to (**a**) at $\omega _{pe}t=1178$.

**Figure 6:**
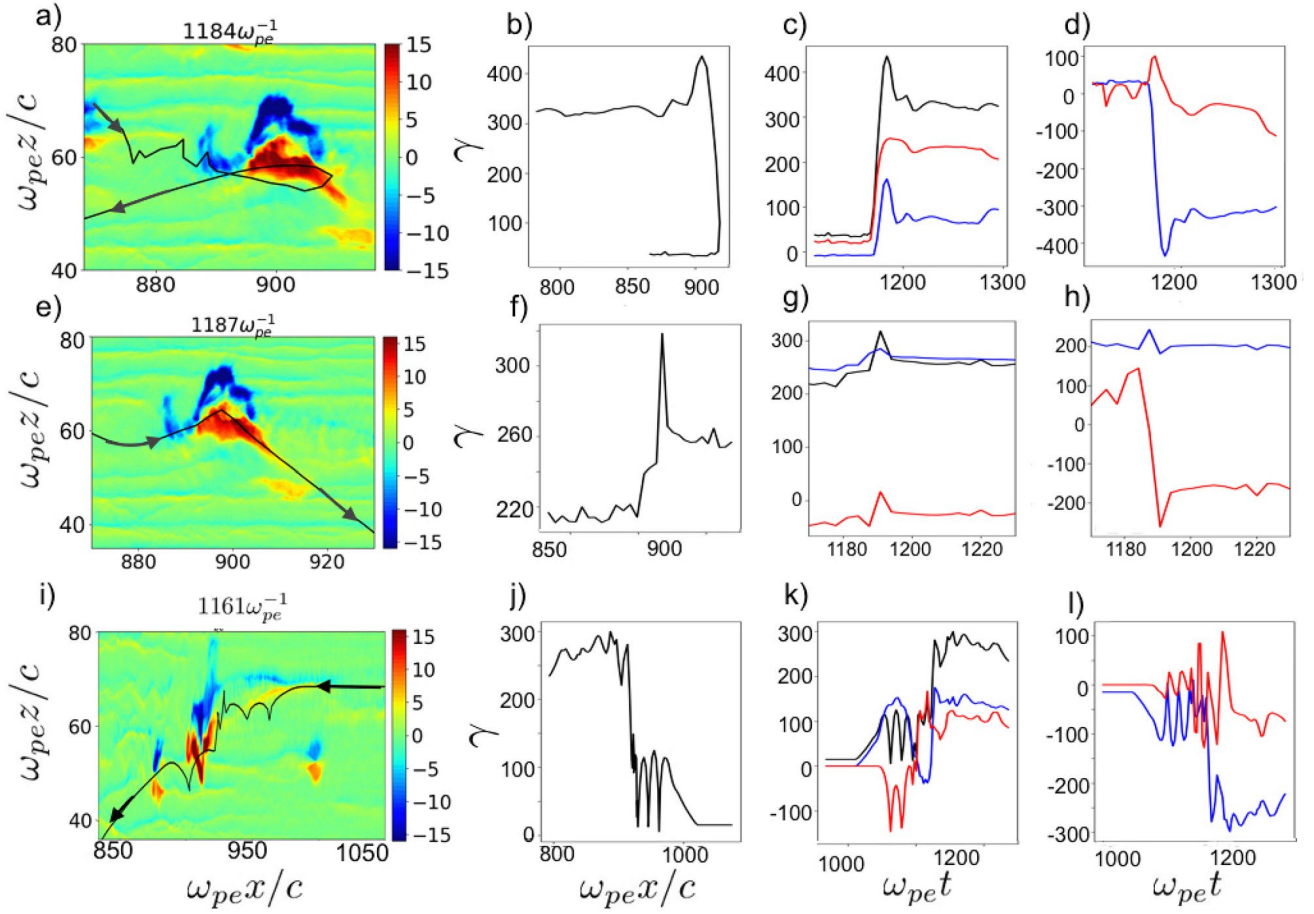
Left column (**a**,**e**,**i**) shows the distributions of transverse electric fields of MV. The trajectories of energized electrons are superimposed and shown by black color. The middle column (**b**,**f**,**j**) shows the electron energy as a function of *x*. The third column (**c**,**g**,**k**) illustrates electron energies (black) and work done by longitudinal (blue) and transverse (red) electric fields on the particle as a function of time. The fourth column (**d**,**h**,**i**) shows the longitudinal (blue) and transverse (red) momentum ($p_x/mc, p_z/mc$) of electrons as a function of time

As we mentioned above electron energization (acceleration and heating) in the upstream of relativistic collisionless ion/electron shocks is governed by stochastic diffusion. In the absence of binary collisions its role plays particles interaction with EM fields of magnetic vortices. Such effective collisions enable particle acceleration to ultra-relativistic energies via a Fermi-like acceleration mechanism which is similar to acceleration at the front of shock wave, but it results from particle scattering in the interaction process with magnetic fluctuations (self-generated magnetic vortices). This stochastic process of acceleration and heating is characterized by the existence of non-thermal tail of the electron distribution function and considerable part of kinetic energy carried by the non-thermal particles. A power law distribution of accelerated electrons for electron-ion collisionless shock with no external field has been revealed in 2D and 3D PIC simulations with indexes being in the interval range $p=2.1- 2.7$ for Fermi^6,37,38^ and Fermi-like acceleration type^5,39,40^. The power law index revealed in the simulation, $p=2.1\pm 0.1$ (see fit in Fig. 4) for the upstream electron spectrum is consistent with the previous simulations for electron-ions shocks. During acceleration process the magnetic dipoles appear and annihilate periodically, at the same time the tail in electron energy spectrum is stable for relatively long interval of time ($\omega _{pe} t> 500$). Thus, the supra-thermal electron tail is formed as a result of the interaction with magnetic fluctuations (self-generated magnetic vortices) developed at nonlinear stage of filamentation in the upstream.

### Electron energization mechanism

In the following, we will discuss electron energization scenarios in interaction with MVs resulting in energized electron tail in electron energy distribution in Figs. 3e and 4. To understand the details of particle acceleration in evolving fields of MV, we tracked the detailed motion of the electrons from the tail of the energy spectrum. The work done by each component of electric field on each particle is calculated throughout the simulation: $W_i=\int _0^t dt' (p_i/\gamma m_{e,i})(\pm e E_i)$, where $i=x,y,z$. Three distinct types of energized electrons were observed in interaction of electrons with MV. All groups of electrons show stochastic behavior of trajectories in the MV fields. Our analysis of many MVs in the upstream of the shock indicates that $\sim 90 \%$ of the energized electron flow from non thermal tail of energy spectrum move towards (or return) to the shock region after gaining energy from electric fields of MV. Figure 5 shows the electron phase distribution averaged over transverse size of the MV at $\omega _{pe}t=1178$ (see Fig. 3d,e). Energetic electrons from the tail of the energy spectrum have negative longitudinal momentum. The longitudinal momentum distribution of the electrons (Fig. 5) averaged over the box shown in Fig. 3b, illustrates the population of electrons moving towards (or returning) to the shock transition region. The peak around $p_x\sim -220m_e c$, shown in Fig. 5b corresponds to the population of electrons shown in Figs. 3d, 5a. A few percent of counter stream electrons from the non-thermal tail of energy spectrum are pre-accelerated before interaction with MV. These electrons gain some extra energy and continue towards the upstream after leaving MV. The third type of energetic electrons are from the incoming electron flow. These electrons trap in linear stage of MV formation and move with MV until annihilation. They gain energy from the electric fields of MV and leave MV while move towards shock transition region.

We start with the first type of electron energization mechanism: a typical counter stream electron moving along $+x$ direction, towards the upstream, experiences the magnetic force of $ev_zB_y (-\hat{\mathbf{x }})$ ($v_z<0, B_y>0$ in this case (Fig. 6a–d)) along $-x$-direction which is larger than the electric force $-eE_x$ (note that $|E_x| <|E_z|,|B_y|$, See Fig. 2 and supplementary material 1). Supplementary material 1 clearly shows the kick-like energization scenario for such electrons during their interaction with nonlinear MV. This causes the electron to abruptly turn and move in the opposite direction towards shock transition region. Meanwhile it gains the energy while moving in the positive lobe of the longitudinal electric field of MV during reflection. The energy gain of electron from longitudinal electric field continues during reflection of the electron ($-eE_xdx>0,(E_x>0, dx<0)$). At the same time the electron gains energy as it moves in transverse electric field ($-eE_z dz, E_z>0,dz<0$ Supplementary material 1), and therefore the energy of the electron increases significantly.

As the electron passes the center of the MV and moves towards the negative lobe of longitudinal electric field of the dipole, it loses a fraction of its energy and finally, the electron leaves the MV at its final stage with energy gain of more than an order of magnitude larger than its original energy and moves towards the shock region. A typical trajectory of such energetic electron from the tail of the energy spectrum is illustrated in Fig. 6a overlaid on transverse electric field distribution at $\omega _{pe}t=1132$ showing the return of the electron towards shock region. Figure 6b shows the energy gain of the electron plotted as a function of *x*, showing the energy gain and return of electron during this process. The evolution of total energy and work done by the electric field components is plotted in Fig. 6c. The work done by both longitudinal and transverse electric field of the MV is leading to the energization of such electrons. Figure 6 shows energetic electron behavior from tail of the energy spectrum corresponding to energization mechanism discussed here. Figure 6a shows the energy gain of such electrons as a function of longitudinal direction (*x*), showing the return of these electrons while gaining a large amount of energy. We can see that electrons interacting with nonlinear stage of MV, where fluctuating fields reach their largest magnitude, gain more energy than the electrons interacting with MV at earlier times, when the fields are still growing. Figure 6b,c show the energy gain time evolution and longitudinal momentum of such particles, confirming their return to shock region. A characteristic behavior of these energetic electrons is that they return to the shock region after interaction with MV.

The second type of energized electrons are the pre-accelerated counter stream electrons moving toward the upstream of the shock (Fig. 6e–h). These electrons already have large energies ($\gamma _{initial}>160$ for the typical electron in Fig. 6e moving towards the upstream prior interacting with nonlinear MV. The transverse component of magnetic Lorentz force ($-ev_{x} B_{y}(\hat{\mathbf{z }})$) kicks the electron out of MV. For the typical electron shown in Fig. 6e $v_{x}>0, B_{y}>0$, therefore the magnetic Lorentz force is along $-\hat{\mathbf{z }}$ and the electron is kicked out of MV (see Supplementary material 2). The electron loses energy while moving into MV ($E_{z}>0,~dz>0$) (Fig. 6e). Then the magnetic force of MV divert the electron, and the electron gains energy while moving out of MV ($E_{z}>0, ~dz<0$). It then continues towards the upstream. Figure 6e shows the trajectory of such electron overlaid on electric field distribution at $\omega _{pe}t=1187$, showing typical electron continue towards the upstream after interaction with fluctuating fields of MV at its nonlinear stage. Figure 6f shows the electron energy as a function of *x* which shows some energy gain for electron before leaving MV. Figure 6g shows that most of the energy gain is from transverse electric field.

The third type of energetic electrons from the tail of the energy spectrum are the incoming electron flow. The incoming electron enters the upstream of the shock and quickly traps in the electric field of MV during linear stage of MV formation. The longitudinal electric field of MV traps the electron, so the electron moves with MV towards shock transition region. At the same time, fluctuating electric and magnetic fields of MV grow significantly. The trapped electron reflects from one lobe to the other due to transverse magnetic Lorentz force. Its energy oscillates rapidly between 15 and $100 m_ec^2$ for typical electron shown in Fig. 6i (Supplementary material 3). Finally at the final stage of MV, the electron leaves MV while gaining energy mostly from transverse electric field and continues towards shock transition region (Fig. 6i).

We never observed incoming electrons returning to the upstream for obvious reason: the incoming electron flow has mostly longitudinal momentum, therefore the longitudinal force of $-ev_zB_y \hat{\mathbf{x }}$ is not large enough to return the electron to the upstream (because $v_z$ is very small or zero). In addition, the longitudinal electric field force of $-eE_x \hat{\mathbf{x }}$ helps electrons to trap in the MV and move with MV. Chaotic (stochastic) motion of an electron in a complex field of a magnetic vortex described above is accompanied by diffusion in the momentum space; this diffusion is induced by stochasticity developing in a combined EM field. The global time-varying electrostatic potential and inductive electric fields contribute to the electron heating process. In general, the mechanism of stochastic acceleration in complex fields of MV is somewhat similar to that proposed in Ref.^28^. Balikhin et al. ^28^ studied stochastic acceleration of electrons in fairly steep gradients of electrostatic and magnetic fields. In the indicated case, the transition to stochastic dynamics happens in the regular structure of the EM field. However, the structure of the MV fields are more complex and nonstationary (and EM fields are fluctuating especially in the final stage of MV evolution), meanwhile electron dynamics is strongly relativistic.

## Discussion

We studied the process of electron energization in interaction with MVs in the upstream of electron/ion shock using 2D PIC simulations. MVs are self-consistently generated in the upstream of electron/ion collisionless shock^19^. The fields of MVs grow as they move toward shock region. Electron energization happens as the electrons (counter-stream and incoming flow) interact with electric fields of MVs in the upstream. Three Fermi-like scenarios of stochastic electron energization were discussed: large fields of MVs can return counter stream electrons towards shock, meanwhile electrons gain significant amount of energy during interaction with electric fields of MV. The second energization mechanism happens when counter stream electrons receive a kick during interaction with MVs due to Lorentz force. The electrons gain energy while being kicked (kick-acceleration) due to sharp electric field gradient. The energized electrons continue moving towards overlapping region. The third scenario is energization of incoming cold flow of electrons. Incoming electron flow can trap in the electric field of the MV in its linear stage. The electrons move with MV as fields of MV grow and finally leave MV while gaining energy as MV annihilates. These electron energization processes happen on time scale of MV formation and evolution and does not require long times as it is needed in shock front. Note that ‘kick’ electron acceleration means particle stochastic dynamics in the fields characterized by the sharp gradients of combined electric and magnetic fields of MV. Electrons accelerated in the process of interaction with the MVs are potentially able to become more energetic by multiple shock front crossings, i.e., Fermi acceleration mechanism in shock front.

Stochastic acceleration results in generation of non-thermal particles that form a power law spectrum. The energy of non-thermal electrons from the tail of spectrum changes with time due to stochastic character of Fermi-like electron acceleration in electric and magnetic fields of evolving MV. After the shock is formed, the upstream forms and expands, a power-law of supra-thermal particles is formed, which saturates rather quickly by $t\omega _{pe}=600$ and stay stable with power law index of $p\approx 2.1$.

In reality, magnetic vortices are three dimensional entities, where the current flow will be along its central axis and electron current flows around the cavity and forms a spheroidal or ellipsoidal shell. It is expected that magnetic vortices would be self-generated in the upstream of electron/ion shocks. However, due to the additional degree of freedom, MV lifetime might be shorter than the 2D calculations which can potentially change accelerated electron population in interaction with the MVs.

The focus of our work is on electron energization in the process of interaction with MVs. We consider the described dynamics as a new interesting stage of electron energization to be very important for formation of non-thermal spectra of particles, since the electron acceleration strongly correlates with the growth and dissipation of MVs. We believe these electrons are potentially able to become more energetic in the process of multiple crossing of the shock wave front at long stages of interaction due to the Fermi process^20–25^.

It is worth mentioning that high power laser facilities provide a unique opportunity for laboratory experiments using plasma flows driven by high energy laser systems which opens a new era in astrophysics and space exploration. Laboratory experiments open the door to investigate the electron-ion sub-relativistic and relativistic collisionless shocks, magnetic field generation and amplification, magnetic reconnection, and particle acceleration in a short temporal and limited spatial scale via laser-plasma interactions^41–44^. Energy transfer from fast ion flow to electromagnetic fields and fast particles (electrons and ions), at a time scale much shorter than electron-ion collisional energy exchange time can be modelled in laboratory conditions. Simulations are scalable to astrophysical conditions with the plasma densities of the order of a few particles per cm$^3$ to the laboratory scale with laser-produced plasmas with densities about 18-20 orders of magnitude higher^45^. Reaching the collisionless regime also allows the instability dynamics to be described by dimensionless parameters and scaled between laboratory and astrophysical systems.

## Supplementary Information

Supplementary Information 1.

Supplementary Information 2.

Supplementary Information 3.

Supplementary Information 4.
