# Supplementary material for: Electron energization dynamics in interaction of self-generated magnetic vortices in upstream of collisionless electron/ion shocks
Source: Sci Rep. 2022 May 5;12:7327. doi: 10.1038/s41598-022-11163-2 (PMC9072358; doi:10.1038/s41598-022-11163-2)
Supplement: Supplementary file 1 — Supplementary Information 1. [file 41598_2022_11163_MOESM1_ESM.pdf]

Legends for Supplementary Videos:

Supplementary Video 1 (track_76.mov): trajectory of a sample counter stream electron in interaction with evolving fields of magnetic dipole corresponding to electron energization scenario 1 in the paper.

Supplementary Video 2(track_70.mov) : trajectory of a sample counter stream electron in interaction with evolving fields of magnetic dipole corresponding to electron energization scenario 2 in the paper.

Supplementary Video 3 (track_74.mov): trajectory of a sample incoming electron in interaction with evolving fields of magnetic dipole corresponding to electron energization scenario 3 in the paper.
